# Case Report: A novel likely pathogenetic variant of the *MEN1* gene in multiple endocrine neoplasia type 1

**DOI:** 10.3389/fendo.2025.1551087

**Published:** 2025-05-12

**Authors:** Mengli Sun, Xinxia Chang, Xianen Huang, Liangmiao Chen, Mengmeng Peng, Xiqiang Zhong

**Affiliations:** ^1^ Department of Endocrinology, Third Affiliated Hospital of Wenzhou Medical University, Wenzhou, Zhejiang, China; ^2^ Department of Endocrinology, Zhongshan Hospital, Fudan University, Shanghai, China

**Keywords:** multiple endocrine neoplasia type 1, variant of uncertain significance, mutation, pancreatic neuroendocrine tumor, parathyroid adenoma

## Abstract

**Background:**

Multiple endocrine neoplasia type 1 (MEN1) is a rare disease caused by mutations in the oncosuppressor gene *MEN1* and characterized by co-occurrence of tumors of the parathyroid gland, pancreas, and pituitary gland. The clinical manifestations of MEN1 are varied, and misdiagnosis is common. The life expectancy of patients with untreated MEN1 is short. Here, we report a case of a 50-year-old patient with recurrent urinary calculi for more than 10 years who had a pancreatic neuroendocrine tumor and parathyroid adenoma. The patient received a definitive diagnosis of MEN1. We analyze his clinical characteristics and describe our approach to management.

**Case Presentation:**

Laboratory tests showed high parathyroid hormone (PTH), high blood calcium, and low blood phosphorus levels and increased excretion of urinary calcium. Immunohistochemical analysis showed loss of menin expression in pancreatic tumor tissues. Testing of the *MEN1* gene revealed a variant in exon 9 (c.1257_1268del, p.lle420_Trp423del).

**Conclusion:**

The patient’s clinical characteristics combined with the testing of the *MEN1* gene, it implied the variant was a novel likely pathogenetic variant. For patients with recurrent urinary stones, we recommend measuring blood calcium and PTH, and if there are abnormalities, screening other endocrine glands to exclude the possibility of MEN1.

## Introduction

Multiple endocrine neoplasia type 1 (MEN1) is a rare autosomal dominant hereditary disease with an estimated global prevalence of 3-10/100,000 (1). MEN1 is characterized by co-occurrence of tumors of the parathyroid gland, pancreas, and pituitary gland. Less frequently patients may develop other endocrine tumors, including adrenal cortical tumors and thymic and bronchial carcinoids, and non-endocrine tumors, including meningiomas, facial angiofibromas, and cutaneous lipomas (2).

MEN1 is associated with a mutation in the *MEN1* gene, which is located on chromosome 11q13 and encodes a 610 amino acid protein, known as menin (3). The clinical manifestations of MEN1 are varied, and misdiagnosis is common. Patients with untreated MEN1 have a 50% probability of death by age 50 years, with 50–70% of patients with MEN1 dying from malignant progression or sequelae (4–8). Fortunately, prompt treatment can extend life expectancy in this patient population (8, 9); consequently, early diagnosis and timely treatment are essential.

A clinical diagnosis of MEN1 is established by the presence of one of three criteria: the occurrence of two or more major MEN1-associated endocrine tumors; the occurrence of one of the MEN1-associated tumors in a first-degree relative; or the identification of a pathogenic or potentially pathogenic *MEN1* gene germline mutation, regardless of symptoms (10). A MEN1 diagnosis should be confirmed by genetic testing.

Here, we report a patient with a clinical diagnosis of MEN1. Immunohistochemical analysis demonstrated a lack of menin expression in pancreatic tumor tissue. Subsequent genetic testing revealed that a variant of uncertain significance was detected in the *MEN1* gene, which, combined with the patient’s clinical presentation and immunohistochemical analysis, suggested this may be a novel likely pathogenetic variant of the *MEN1* gene. This case report provides a reference for the early clinical diagnosis of MEN1.

## Case presentation and diagnostic assessment

**Figure 1:**
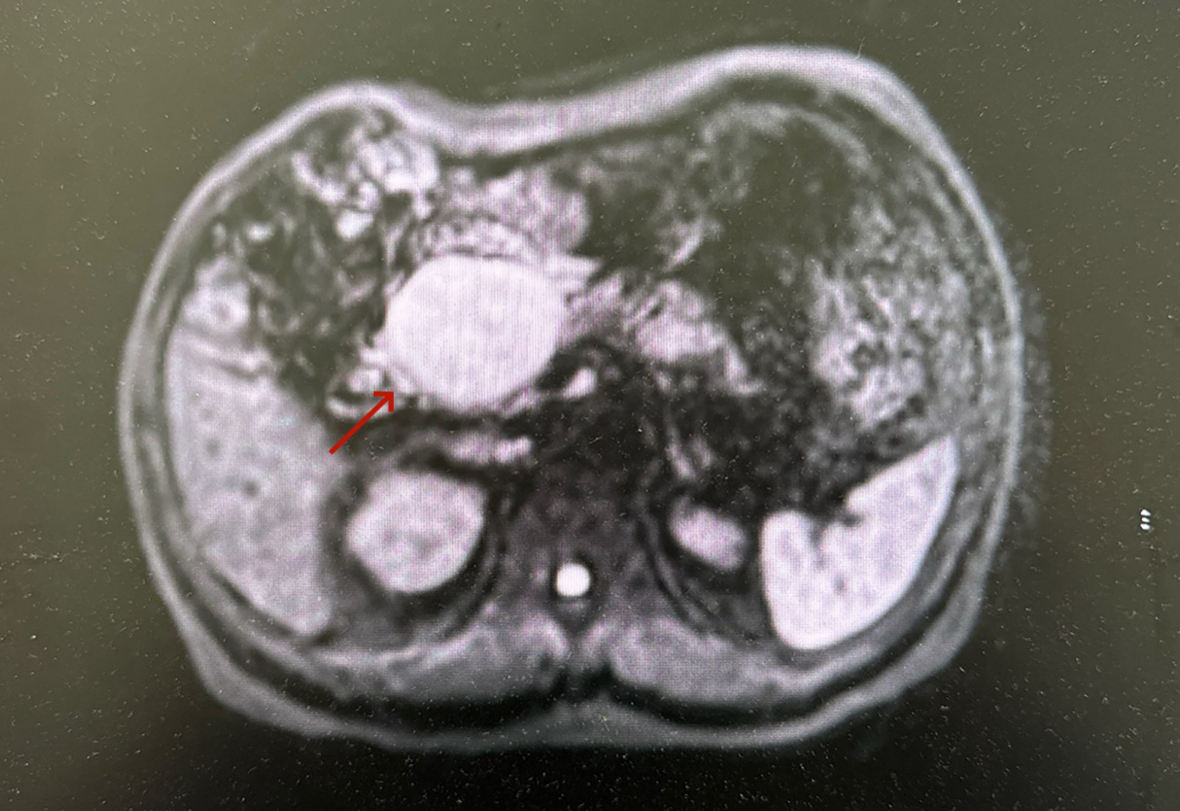
MRI showed a circular heterogeneous focal lesion on the pancreatic head, with a low signal on T1WI, and a high signal on T2WI and DWI. The lesion was approximately 50*58 mm in size, with uneven enhancement.

**Figure 2:**
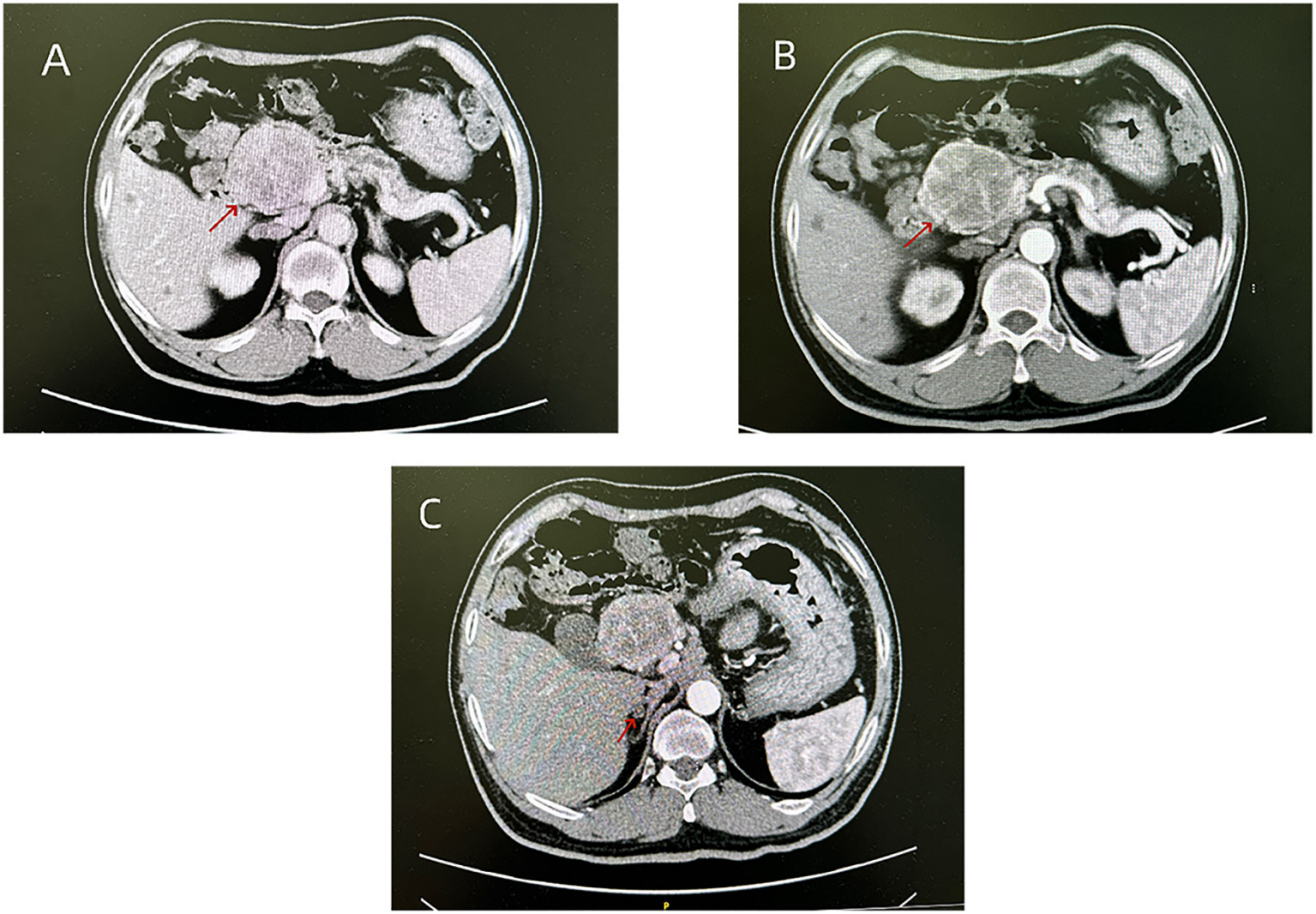
Vascular CT showed a 53*58 mm nodule on the pancreatic head, with uneven enhancement: **(A)** Venous phase, **(B)** Arterial phase; CT showed low density nodules in the right adrenal gland **(C)**.

A 50-year-old male with recurrent urinary calculi for more than 10 years that were treated with extracorporeal lithotripsy when attacks were severe, presented to our hospital with upper abdominal pain that had persisted for 1 month. Computed tomography (CT) and magnetic resonance (MRI) examinations conducted in a local hospital suggested a pancreatic tumor. Enhanced MRI and magnetic resonance cholangiography (MRI+MRCP) in our hospital showed a high probability of a 50*58 mm pancreatic neuroendocrine tumor (pNET) in the pancreatic head (**Figure 1**). Vascular enhanced CT also showed a lesion in the pancreatic head, suggesting the possibility of a pNET (**Figures 2A, B**). Physical examination revealed no abnormality when the patient was admitted to our department. Family medical history revealed a brother and sister with urinary calculi, but the patient’s parents and brother had passed away, so medical records were not available. The patient’s son did not have similar manifestations. Further follow-up on the patient’s medical history showed he had undergone surgery for perforated gastric ulcers 5 years earlier. Written informed consent for the publication of this case report was obtained from the patient.

On September 3, 2024, the patient underwent extensive radical resection of the pancreatic tumor under general anesthesia. Pathology showed a tumor composed of epithelioid cells arranged in nests and flakes, 1–2 mitoses per 10HPF, and a Ki-67 index of 3%, reporting a diagnosis of Grade 2 pNET. Immunohistochemistry was positive for CgA (partial +), SSTR2 (100%+++), SSTR5 (100%++), CD56 (+), SYN (+), P53 (10%+), CD99 (+), and CD19 (+), and showed loss of menin expression. Considering the patient had experienced recurrent urinary calculi for more than 10 years, MEN-1 could not be excluded, and the patient was referred to the endocrinology department.

**Figure 3:**
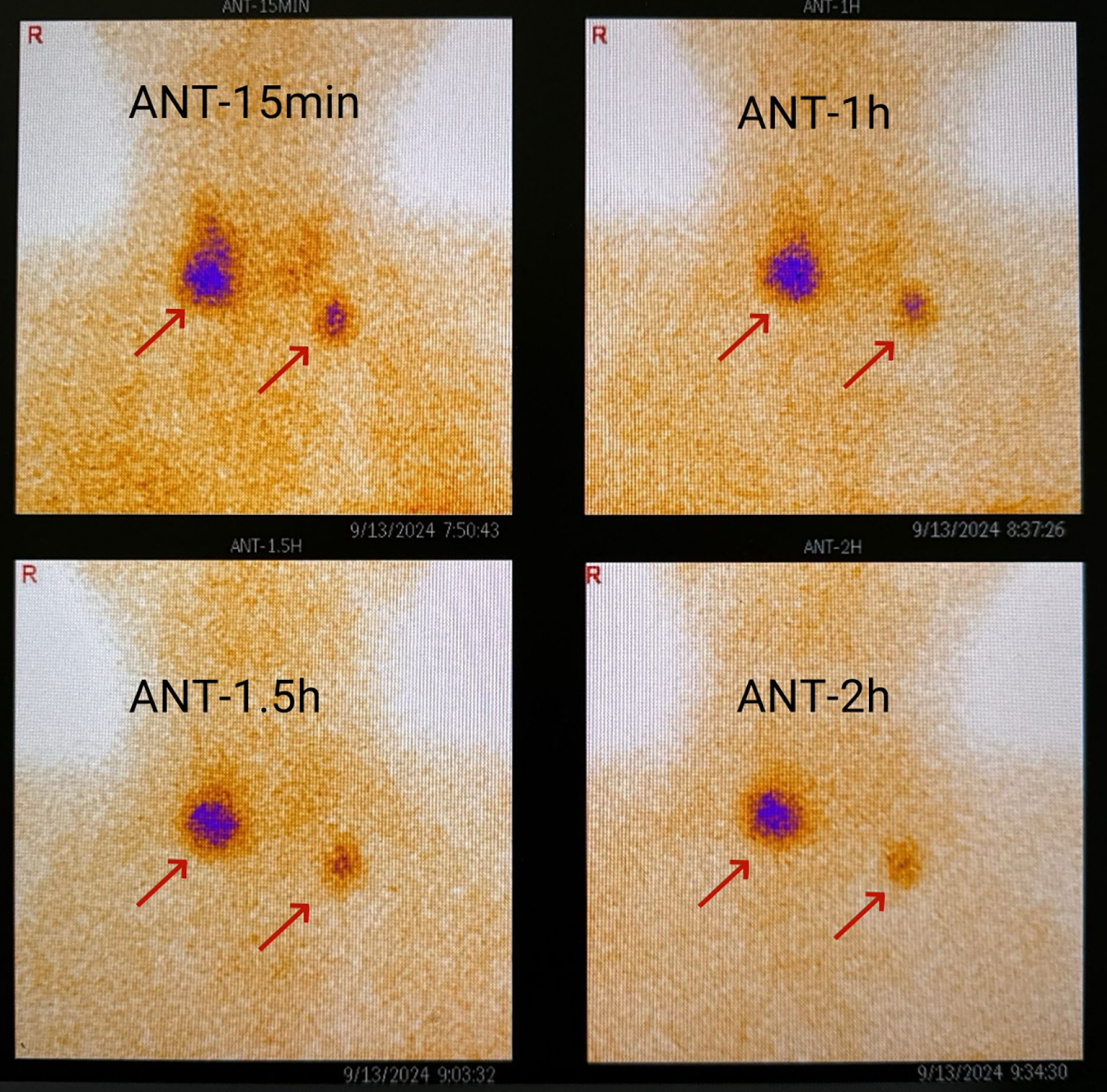
Parathyroid nuclide imaging showed abnormal low-density nodules in the right inferior pole of the thyroid gland and the left clavicular region at 15 minutes, 1 hour, 1.5 hours, and 2 hours after intravenous injection of 99mTc-MIBI, with sizes of 32.4×20.3 mm and 13.1×8.9 mm, respectively.

Results of the patient’s laboratory tests during hospitalization are shown in **Table 1**. Serum calcium level was 3.93 mmol/L (normal range: 2.15-2.55 mmol/L), serum phosphate level was 0.35 mmol/L (normal range: 0.90-1.34 mmol/L), serum parathyroid hormone (PTH) level was 1081 pg/m L (normal range: 15.0-65.0 pg/m L), and urine calcium was 13.05 mmol/24 h (normal range 2.5-8.0 mmol/24 h). Ultrasound of the neck showed a parenchymal mass below the inferior pole of the right lobe of the thyroid gland, which may have been of parathyroid origin. Radionuclide imaging of the parathyroid gland showed a concentrated shadow of technetium-99 m-methoxyisobutylisonitrile (MIBI) in the lower right lobe of the thyroid and in the left clavicular region (**Figure 3**), indicating hyperfunctional parathyroid tissue. Based on these results, the patient was diagnosed with primary hyperparathyroidism. Bone mineral density on dual-energy X-ray showed the T-score of the lumbar spine (L1-4) was -4.8, and the T-score of the left femoral neck was -2.8, meeting the diagnostic criteria for osteoporosis. Abdominal CT showed no urinary tract stones. On November 15, 2024, the patient underwent right parathyroidectomy plus left central lymph node dissection for primary hyperparathyroidism. The postoperative pathological diagnosis was parathyroid adenoma. The resected lymph nodes showed fibrous adipose tissue. The surgery was successful, with a preoperative PTH level of 1081 pg/mL and a PTH level at postoperative 1.5 hours of 94.1 pg/mL.

**Table 1:** Laboratory tests.

Parameter	Values	Reference range
Serum albumin, g/L	35	35–55
Serum calcium, mmol/L	3.93	2.15–2.55
Serum phosphate, mmol/L	0.35	0.90–1.34
Alkaline phosphatase, U/L	143	45–125
PTH, pg/mL	1081	15.0–65.0
25-hydroxyvitamin D, nmol/L	18.0	>50
24 hours Urine calcium, mmol/24h	13.05	2.5–8.0
urinary calcium/creatinine, mmol/mmol	1.78	0–0.57
Serum creatinine, umol/L	92	44–115
HbA1c, %	5.6	4–6
Fasting serum glucose, mmol/L	4.8	3.9–5.6
ACTH (8AM), pg/mL	18.5	7.2–63.3
ACTH (4PM), pg/mL	11.1	–
ACTH (12PM), pg/mL	6.6	–
Cortisol (8AM), nmol/L	381.0	133.0–537.0
Cortisol (4PM), nmol/L	258	–
Cortisol (12PM), nmol/L	91.7	–
TSH, uIU/mL	1.76	0.27–4.2
Free T3, pmol/L	3.7	3.1–6.8
Free T4, pmol/L	15.6	12.0–22.0
Calcitonin, pg/mL	1.2	0–9.52
Prolactin, mIU/L	326	131–647
LH, mIU/mL	3.3	1.7–8.6
FSH, mIU/mL	4.1	1.5–12.4
Estradiol, pmol/L	34.2	41.4–159
Testosterone, nmol/L	9.3	6.68–25.7
Growth hormone, ng/mL	0.3	<1.0
IGF, ng/mL	43.2	75.0–307
Gastrin, pg/mL	27.30	13.0–115.0
Insulin, uU/mL	6.9	2.6–24.9
C-peptide, g/mL	1.32	1.1–4.4
PRA, ng/mL/h	0.151	0.250–5.820
Aldosterone, pg/mL	<12.5	30–160
Sulfuric DHEA, umol/L	0.237	1.2–8.98
metanephrine, pg/mL	46.0	<96.6
Normetanephrine, pg/mL	119.2	<163.0
3-methoxytyramine, pg/mL	<10.0	<21.7

Considering that patients with MEN1 may have gastrinoma, gastrin levels were measured and found to be 27.30 pg/mL (normal range:13.0-115.0 pg/mL), so a diagnosis of gastrinoma was not supported. Enhanced abdominal CT suggested a right adrenal adenoma (**Figure 2C**). The patient’s laboratory tests (**Table 1**) indicated a cortisol rhythm and normal levels of aldosterone, renin and the intermediate metabolites of catecholamine, with no evidence to support a functional adrenal adenoma. In addition, the patient’s pituitary MRI showed no space occupying lesion, and the patient’s thyrotropin (TSH), adrenocorticotropin (ACTH), luteinizing hormone (LH), follicle-stimulating hormone (FSH), and prolactin levels were all in the normal range (**Table 1**).

Based on these findings, the patient received a definitive clinical diagnosis of MEN1; therefore, genetic testing of the *MEN1* gene was performed. Whole-exome sequencing was conducted with New High-throughput Sequencing Technology (NGS) based on the MGISEQ-2000 Platform. This assay did not specify candidate genes, but performed whole-exome-wide analysis, with overall coverage of more than 95%. The T/SZGIA 4–2018 genetic testing reporting standard was used with hg38 as the reference genome. Deep whole-exome sequencing showed genetic variation c.1257_1268del on exon 9 of chromosome 11(chr 11:64572589-64572600), which resulted in an alteration of the amino acid sequence p.lle420_Trp423del (NM_001370251.2: exon 9). This heterozygous mutation occurred between the isoleucine at position 420 and the tryptophan at position 423 of the encoded protein. According to the Exome Sequencing Project (ESP) database, the 1000 Genomes Project (1KGP) database, the Genome Aggregation Database (gnomAD), the Human Gene Mutation Database (HGMD) and ClinVar, this mutation has a population frequency of 0. Considering the patient’s clinical symptoms, related disease characteristics and genetic testing, and according to the 2015 American College of Medical Genetics and Genomics (ACMG) guidance for the interpretation of sequence variants, the variant detected in this patient may have been related to the clinical manifestations, and it was judged to be a likely pathogenic variant, according to the available evidence. Given that MEN1 transmission is autosomal dominant, we recommended the patient’s first-degree relatives undergo genetic testing. Unfortunately, the patient’s sister was not willing to undergo genetic testing, and only chose to undergo blood glucose, blood calcium, blood phosphorus and PTH screening in a local hospital, and the results of these tests were normal. The patient’s son is currently studying abroad and has promised to undergo genetic screening as soon as possible after returning home.

After surgery, the patient was followed up in our hospital and at the local hospital. Blood calcium, blood phosphorus, PTH, blood glucose and insulin levels were all in the normal range, and there was no discomfort.

## Discussion

MEN1 is an autosomal dominant genetic disorder characterized by a high degree of disease penetrance after age 55 years. Age of symptom onset can range from 5 to 81 years (1, 10, 11), with 50% of patients showing clinical symptoms by age 20 years, 95% by age 40 years (10), and nearly 100% by age 50 years (11). The clinical manifestations of MEN1 vary depending on the tumor site and the hormone secreted by the tumor, and MEN1 may be misdiagnosed. In the present case, the patient had recurrent urinary stones for more than 10 years and had been treated with extracorporeal lithotripsy, but the attending physician had never considered the possibility of MEN1. Upon admission to our hospital, the patient had high PTH and blood calcium (1081 pg/mL, 3.93 mmol/L, respectively) levels and a large pancreatic tumor (50*58 mm). After resection of the pNET and hyperfunctional parathyroid tissue, PTH, blood calcium, blood glucose and insulin levels, and levels of other related indicators, were satisfactory. Many tumors associated with MEN-1 are benign; however, the life expectancy in patients with MEN1 is significantly reduced by 15 years compared to the general population (8). Studies with 30 years of follow-up estimate the average life expectancy of patients with MEN1 as 55 years (9, 12, 13). A timely diagnosis of MEN1 is important for the patient, but also for family members, as first-degree relatives have a 50% risk of developing the condition (10).

In our case, the patient had the following disease characteristics: (1) history of recurrent urinary calculi, and both his brother and sister had a history of urinary calculi; (2) previous surgery for gastric ulcer; (3) laboratory tests showed high PTH, high blood calcium and low blood phosphorus levels, and increased excretion of urinary calcium; (4) imaging showed parathyroid tumors, pancreatic tumors, adrenal tumors, and thyroid tumors; (5) postoperative pathological analysis of pancreatic and parathyroid glands suggested a pNET and a parathyroid tumor; and (6) bone mineral density indicated osteoporosis. The patient had severe osteoporosis at the time of his first visit to our hospital, with a T-score of -4.8; this was considered secondary damage caused by long-term hypercalcemia. This case should raise awareness among physicians of the possibility of MEN1 in patients with urinary tract stones, and that timely assessment of blood calcium and PTH levels is essential, allowing early detection of parathyroid adenoma and surgical treatment.

There are many surgical options for parathyroid adenomas (1). The traditional approach was total parathyroidectomy (TPTX) + autologous transplantation, but the risk of permanent postoperative hypoparathyroidism was high (14, 15), and the procedure is no longer advocated (8). The current recommendation is subtotal parathyroidectomy (SPTX), removing 3 or 3.5 parathyroid glands, which offers similar surgical benefits compared to TPTX, but with a lower risk of postoperative adverse effects. Unilateral parathyroidectomy is now also advocated for patients with a single adenoma (1). Outcomes are comparable to SPTX, and a 4-year follow-up showed no signs of hypoparathyroidism. In patients with MEN1, who often have asymmetrical hyperplasia of the parathyroid glands, unilateral parathyroidectomy provides a less aggressive initial resection, allowing a second operation on the previously nonoperative side of the neck, when necessary (16). Another option is to remove one or more glands if they are identified on functional imaging as the cause of high PTH secretion. Although this procedure is simple and does not cause hypoparathyroidism, it has a high recurrence rate compared to TPTX or SPTX. In the present case, the patient underwent right parathyroidectomy, and PTH decreased from 1081 pg/mL before surgery to 94.1 pg/mL at postoperative 1.5 hours. These findings indicate that the treatment for parathyroid adenoma was successful; however, we recommend patient follow-up includes regular evaluation of serum calcium and PTH levels.

This patient was found to have a tumor in the pancreatic head due to abdominal pain. Laboratory tests showed that blood glucose, insulin and gastrin levels were normal, and the pNET was considered non-functional; however, the tumor was resected due to its large size. MEN1-associated non-functional pNETs may have a worse prognosis than other functional tumors such as insulinoma and gastrinoma (17, 18), and pNETs are one of the most common causes of death in patients with MEN1 (2, 18, 19). pNETs associated with MEN1 are often multifocal and biobehaviorally uncertain; therefore, we recommend pancreatic imaging and related blood indicators should be reviewed regularly in this patient. At present, postoperative follow-up showed that the patient is recovering, and reexamination of blood calcium, blood phosphorus, PTH, blood glucose and insulin showed normal levels. The patient and her family were very satisfied with the treatment the patient received.

Abdominal CT of the patient in this case study showed a right adrenal adenoma. Adrenal hormone tests did not show primary hyperaldosteronism and hypercortisolism, therefore, the tumors were considered non-functional. Since there is no consensus on the treatment of MEN1-related nonfunctional tumors, we recommend a watch and wait approach for this patient. Adrenal tumor surgery is required in the following cases: (1) patients with tumors > 4cm in diameter; (2) patients with 1-4cm diameter and atypical or suspicious imaging features; (3) patients with significant tumor enlargement within 6 months (17–19).

Multiple endocrine adenoma, as an autosomal dominant hereditary disease mainly manifested by hyperfunction, is divided into multiple endocrine adenoma type 1, multiple endocrine adenoma type 2 and multiple endocrine adenoma type 4. The disease spectrum of various MEN types is different, but some of the same clinical phenotypes exist. Genetic testing has emerged as an important medical tool to inform clinical decision-making. MEN2 is caused by germ line mutation of the *RET* gene (20–22), MEN4 is related to *CDKI* (*CDKN1B/p27/KIP1*) gene mutations (23, 24), and MEN1 is mainly caused by *MEN1* gene mutations (3).In the first 10 years since the discovery of the *MEN1* gene, 1336 mutations were identified, including 1133 germline mutations and 203 individual cell mutations (25), with the total number of mutations now exceeding 1800 (26). In the present case, a c.1257_1268del variant (p.lle420_Trp423del) was found in the *MEN1* gene, which resulted in an alteration of the amino acid sequence p.lle420_Trp423del (NM_001370251.2: exon 9) between an isoleucine at position 420 and a tryptophan at position 423 of the encoded protein. No reports of this mutation were found in any relevant database, and the variant was judged to be of uncertain significance following ACMG. However, the clinical presentation of this patient supported the diagnosis of MEN1, and immunohistochemistry suggested that menin expression was absent, implying possible pathogenicity of the current variant (c.1257_1268del). The clinical significance of this variant requires further clarification by testing family members, and the pathogenicity of this variant needs to be confirmed *in vitro*. Unfortunately, the patient’s sister has not yet agreed to genetic testing. Notably, the mutation detected in the subject was located in exon 9 of the *MEN1* gene, and one study showed that mutations in exon 2, 9, and 10 of the *MEN1* gene are associated with a higher incidence of malignant tumors (27). Therefore, comprehensive follow-up of this patient is essential. Importantly, the incidence of *CDKN1B* gene mutations in patients with MEN1-related manifestations may be 1% to 4% (28–30). If genetic testing of our patient had not found a mutation in the *MEN1* gene, screening for MEN4 should have been performed.

Although there is improved understanding of the diagnosis and treatment of MEN1, due to the polymorphism of the *MEN1* gene and its lack of genotypic/phenotypic consistency, it is impossible to predict associated tumor types based on the mutated gene, so MEN1 is a challenging disease. We recommend MEN1 is treated by a multidisciplinary team that includes specialists in endocrinology, radiology, gastroenterology, neurosurgery, and genetics. Considering the variable clinical manifestations of MEN1 and the many clinical departments that may encounter MEN1-related diseases, diagnosis may be delayed, so knowledge sharing among medical experts is essential.

## Conclusion

The patient in this case had typical clinical manifestations of MEN1. Genetic testing of the *MEN1* gene showed a variant of uncertain significance. This combined with the patient’s clinical characteristics implied the variant was a novel likely pathogenetic variant. For patients with recurrent urinary stones, we recommend measuring blood calcium and PTH, and if there is an abnormality, screening other endocrine glands to exclude MEN1.

## Data Availability

The original contributions presented in the study are included in the article, further inquiries can be directed to the corresponding authors.

## References

[1] Goudet P Cadiot G Barlier A Baudin E Borson-Chazot F Brunaud L. French guidelines from the GTE, AFCE and ENDOCAN-RENATEN (Groupe d’étude des Tumeurs Endocrines/Association Francophone de Chirurgie Endocrinienne/Reseau national de prise en charge des tumeurs endocrines) for the screening, diagnosis and management of Multiple Endocrine Neoplasia Type 1. Ann Endocrinol (Paris). (2024) 85:2–19. doi: 10.1016/j.ando.2023.09.003 37739121

[2] Brandi ML Gagel RF Angeli A Bilezikian JP Beck-Peccoz P Bordi C. Guidelines for diagnosis and therapy of MEN type 1 and type 2. J Clin Endocrinol Metab. (2001) 86:5658–71. doi: 10.1210/jcem.86.12.8070 11739416

[3] Larsson C Skogseid B Oberg K Nakamura Y Nordenskjöld M. Multiple endocrine neoplasia type 1 gene maps to chromosome 11 and is lost in insulinoma. Nature. (1988) 332:85–7. doi: 10.1038/332085a0 2894610

[4] Goudet P Murat A Binquet C Cardot-Bauters C Costa A Ruszniewski P. Risk factors and causes of death in MEN1 disease. A GTE (Groupe d’Etude des Tumeurs Endocrines) cohort study among 758 patients. World J Surg. (2010) 34:249–55. doi: 10.1007/s00268-009-0290-1 19949948

[5] Geerdink EA van der Luijt RB Lips CJ. Do patients with multiple endocrine neoplasia syndrome type 1 benefit from periodical screening. Eur J Endocrinol. (2003) 149:577–82. doi: 10.1530/eje.0.1490577 14641000

[6] Dean PG van Heerden JA Farley DR Thompson GB Grant CS Harmsen WS. Are patients with multiple endocrine neoplasia type I prone to premature death. World J Surg. (2000) 24:1437–41. doi: 10.1007/s002680010237 11038219

[7] Doherty GM Olson JA Frisella MM Lairmore TC Wells SA Jr Norton JA. Lethality of multiple endocrine neoplasia type I. World J Surg. (1998) 22:581–6. doi: 10.1007/s002689900438 9597932

[8] Gaujoux S Martin GL Mirallié E Regenet N Le Bras M Pattou F. Life expectancy and likelihood of surgery in multiple endocrine neoplasia type 1: AFCE and GTE cohort study. Br J Surg. (2022) 109:872–9. doi: 10.1093/bjs/znac006 35833229

[9] Ito T Igarashi H Uehara H Berna MJ Jensen RT. Causes of death and prognostic factors in multiple endocrine neoplasia type 1: a prospective study: comparison of 106 MEN1/Zollinger-Ellison syndrome patients with 1613 literature MEN1 patients with or without pancreatic endocrine tumors. Med (Baltimore). (2013) 92:135–81. doi: 10.1097/MD.0b013e3182954af1 PMC3727638 23645327

[10] Thakker RV Newey PJ Walls GV Bilezikian J Dralle H Ebeling PR. Clinical practice guidelines for multiple endocrine neoplasia type 1 (MEN1). J Clin Endocrinol Metab. (2012) 97:2990–3011. doi: 10.1210/jc.2012-1230 22723327

[11] Al-Salameh A Cadiot G Calender A Goudet P Chanson P. Clinical aspects of multiple endocrine neoplasia type 1. Nat Rev Endocrinol. (2021) 17:207–24. doi: 10.1038/s41574-021-00468-3 33564173

[12] Norton JA Krampitz G Zemek A Longacre T Jensen RT. Better survival but changing causes of death in patients with multiple endocrine neoplasia type 1. Ann Surg. (2015) 261:e147–8. doi: 10.1097/SLA.0000000000001211 26291955

[13] Casey RT Saunders D Challis BG Pitfield D Cheow H Shaw A. Radiological surveillance in multiple endocrine neoplasia type 1: a double-edged sword. Endocr Connect. (2017) 6:151–8. doi: 10.1530/EC-17-0006 PMC5424776 28298337

[14] Pieterman CR van Hulsteijn LT den Heijer M van der Luijt RB Bonenkamp JJ Hermus AR. Primary hyperparathyroidism in MEN1 patients: a cohort study with longterm follow-up on preferred surgical procedure and the relation with genotype. Ann Surg. (2012) 255:1171–8. doi: 10.1097/SLA.0b013e31824c5145 22470073

[15] Lairmore TC Govednik CM Quinn CE Sigmond BR Lee CY Jupiter DC. A randomized, prospective trial of operative treatments for hyperparathyroidism in patients with multiple endocrine neoplasia type 1. Surgery. (2014) 156:1326–34. doi: 10.1016/j.surg.2014.08.006 25262224

[16] Kluijfhout WP Beninato T Drake FT Vriens MR Gosnell J Shen WT. Unilateral clearance for primary hyperparathyroidism in selected patients with multiple endocrine neoplasia type 1. World J Surg. (2016) 40:2964–9. doi: 10.1007/s00268-016-3624-9 PMC5104782 27402205

[17] Gatta-Cherifi B Chabre O Murat A Vriens MR Gosnell J Shen WT. Adrenal involvement in MEN1. Analysis of 715 cases from the Groupe d’etude des Tumeurs Endocrines database. Eur J Endocrinol. (2012) 166:269–79. doi: 10.1530/EJE-11-0679 22084155

[18] Schaefer S Shipotko M Meyer S Ivan D Klose KJ Waldmann J. Natural course of small adrenal lesions in multiple endocrine neoplasia type 1: an endoscopic ultrasound imaging study. Eur J Endocrinol. (2008) 158:699–704. doi: 10.1530/EJE-07-0635 18426829

[19] Langer P Cupisti K Bartsch DK Nies C Goretzki PE Rothmund M. Adrenal involvement in multiple endocrine neoplasia type 1. World J Surg. (2002) 26:891–6. doi: 10.1007/s00268-002-6492-4 12016472

[20] Donis-Keller H Dou S Chi D Carlson KM Toshima K Lairmore TC. Mutations in the RET proto-oncogene are associated with MEN 2A and FMTC. Hum Mol Genet. (1993) 2:851–6. doi: 10.1093/hmg/2.7.851 8103403

[21] Eng C Smith DP Mulligan LM Nagai MA Healey CS Ponder MA. Point mutation within the tyrosine kinase domain of the RET proto-oncogene in multiple endocrine neoplasia type 2B and related sporadic tumours. Hum Mol Genet. (1994) 3:237–41. doi: 10.1093/hmg/3.2.237 7911697

[22] Margraf RL Crockett DK Krautscheid PM Seamons R Calderon FR Wittwer CT. Multiple endocrine neoplasia type 2 RET protooncogene database: repository of MEN2-associated RET sequence variation and reference for genotype/phenotype correlations. Hum Mutat. (2009) 30:548–56. doi: 10.1002/humu.20928 19177457

[23] Polyak K Lee MH Erdjument-Bromage H Koff A Roberts JM Tempst P. Cloning of p27Kip1, a cyclin-dependent kinase inhibitor and a potential mediator of extracellular antimitogenic signals. Cell. (1994) 78:59–66. doi: 10.1016/0092-8674(94)90572-X 8033212

[24] Hengst L Reed SI. Translational control of p27Kip1 accumulation during the cell cycle. Science. (1996) 271:1861–4. doi: 10.1126/science.271.5257.1861 8596954

[25] Lemos MC Thakker RV. Multiple endocrine neoplasia type 1 (MEN1): analysis of 1336 mutations reported in the first decade following identification of the gene. Hum Mutat. (2008) 29:22–32. doi: 10.1002/humu.20605 17879353

[26] Falchetti A. Genetics of multiple endocrine neoplasia type 1 syndrome: what’s new and what’s old. F1000Res. (2017) 6:1-10. doi: 10.12688/f1000research PMC5288685 28184288

[27] Bartsch DK Langer P Wild A Schilling T Celik I Rothmund M. Pancreaticoduodenal endocrine tumors in multiple endocrine neoplasia type 1: surgery or surveillance. Surgery. (2000) 128:958–66. doi: 10.1067/msy.2000.109727 11114630

[28] Agarwal SK Mateo CM Marx SJ. Rare germline mutations in cyclin-dependent kinase inhibitor genes in multiple endocrine neoplasia type 1 and related states. J Clin Endocrinol Metab. (2009) 94:1826–34. doi: 10.1210/jc.2008-2083 PMC2684477 19141585

[29] Georgitsi M Raitila A Karhu A van der Luijt RB Aalfs CM Sane T. Germline CDKN1B/p27Kip1 mutation in multiple endocrine neoplasia. J Clin Endocrinol Metab. (2007) 92:3321–5. doi: 10.1210/jc.2006-2843 17519308

[30] Molatore S Marinoni I Lee M Pulz E Ambrosio MR degli Uberti EC. A novel germline CDKN1B mutation causing multiple endocrine tumors: clinical, genetic and functional characterization. Hum Mutat. (2010) 31:E1825–35. doi: 10.1002/humu.v31:11 PMC3051264 20824794

